# Cephalotripsy without Traction Versus the Caesarean Operation

**Published:** 1860-07

**Authors:** 


*Cephalotripsy without Traction versus the Cæsarean Operation.*—M. Pajot, professor of clinical obstetricy at the Paris Faculty, has just substituted the former for the latter operation, in a case where, from rickets, the antero posterior diameter measured but one inch and two-thirds. M. Pajot considers that the danger of cephalotripsy consists in the tractions which are generally made after the head is broken up, as points of bone easily tear the soft parts of the mother. He is in the habit of crushing as completely as possible, and then leaving the uterus to expel the fœtus by its contractions. This method is even carried out as regards the body of the child. In the present case the success was complete.

